# Editorial: Innovating public mental health: integrating digital therapeutics for comprehensive mood disorder management

**DOI:** 10.3389/fpsyt.2026.1989479

**Published:** 2026-10-15

**Authors:** Jun Ji, Yantao Ma

**Affiliations:** 1 College of Computer Science and Technology, Qingdao University, Qingdao, China; 2 Beijing Wanling Pangu Science and Technology Ltd., Beijing, China; 3 National Health Commission (NHC) Key Laboratory of Mental Health (Peking University), Peking University Sixth Hospital, Peking University Institute of Mental Health, National Clinical Research Center for Mental Disorders (Peking University Sixth Hospital), Beijing, China

**Keywords:** artificial intelligence, digital mental health, digital therapeutics, implementation science, mood disorders, public mental health, suicide prevention

## Introduction

Digital therapeutics have a potential role in public mental health, but their value for mood disorders depends on how they contribute to care. A meta-analysis of 176 randomized trials found small average benefits of smartphone interventions for depressive and generalized anxiety symptoms (1). A 2026 evidence map across digital therapeutics authorized by the US Food and Drug Administration found substantial variation in publicly available clinical evidence, reinforcing the need to assess each product and indication separately (2). This Research Topic spans digital interventions and the assessment and implementation research needed to integrate them into care; not every contribution evaluates a digital therapeutic. Software-delivered therapeutic interventions should be distinguished from telemedicine, wellness activities, and assessment tools within the broader digital-care landscape. Its ten articles connect intervention design, implementation, and clinical decision-making, while exposing gaps between these domains. We use integrated, person-centered care as an organizing lens for this heterogeneous collection, which extends beyond diagnosed mood disorders.

## Therapeutic experiences

Brief interventions may offer accessible support during distress. Tu et al. reported reduced depressive symptoms and rumination after five weeks of app-guided mindfulness among people experiencing entrepreneurial failure. Participants with a psychiatric diagnosis history were excluded, so efficacy in diagnosed depression remains unestablished. In two randomized laboratory experiments, Li et al. examined digital mandala coloring after anxiety induction. Self-reported anxiety reduction did not differ significantly between digital and paper coloring, although digital coloring produced greater flow and some physiological improvements. Sliding interaction outperformed a gamified mode; mediation findings implicate flow without establishing a general therapeutic mechanism. Kwak et al. reported acute anxiety, mood, and physiological changes after puppy videos in office workers, but the uncontrolled pre-post design cannot establish efficacy. Together, these studies suggest design opportunities for emotional regulation, while leaving durability, clinical benefit, and added value over existing care unresolved.

## Evidence adoption and reach

Evidence generation must accompany deployment. Li et al. outline a systematic review and meta-analysis of self-guided virtual reality exposure for social anxiety disorder; this protocol supplies no pooled treatment results. In a survey of 483 participants, Nurtsch et al. linked videoconferencing psychotherapy acceptance to expectations and concerns about effectiveness and emotional expression. Acceptance, however, does not establish uptake or sustained engagement. The prevANS recruitment analysis by García-Huércano et al. identified social media and university dissemination as prominent enrollment channels, yet the sample was predominantly female and highly educated. Recruitment success therefore does not establish equitable reach or preventive efficacy. These contributions connect evidence quality with the practical questions of who participates and who remains underserved. Engagement research (3) and co-production evidence (4) support involving intended users throughout development and evaluation.

Recent clinical evidence also distinguishes delivery from therapeutic content. In the SUMMIT trial of 1,230 pregnant or postpartum adults, behavioral activation delivered by nonspecialists was noninferior to specialist delivery, and telemedicine was noninferior to in-person care, for depressive symptoms at three months (5). This supports supervised task-sharing and flexible psychotherapy delivery in that population, not the efficacy of autonomous software treatment.

## Assessment and decision support

The assessment studies identify information needs rather than validated pathways to better outcomes. Yang et al. examined sex-specific associations between three-dimensional facial features and current suicide risk in major depressive disorder. Cross-sectional associations do not establish prospective prediction or clinical utility. Geng et al. associated more severe depressive and anxiety symptoms with higher scale-assessed suicide risk among adolescents with non-suicidal self-injury. Their exploratory network of clinical and social variables does not identify causal treatment targets. These limitations matter because even apparently discriminative machine-learning models can have poor positive predictive value for suicide and self-harm (6). NICE guidance for self-harm care advises against using risk tools to predict future suicide or determine treatment and discharge; assessment should focus on individual needs and safety (7).

Herpertz et al. surveyed physicians about psychiatric dashboard priorities, highlighting current suicidality, previous antidepressant treatment, and illness course. This specifies desired information, not demonstrated patient benefit. Zheng et al. describe a cluster-randomized trial protocol for an artificial intelligence-aided Describe-Investigate-Create-Evaluate workflow addressing behavioral and psychological symptoms of dementia. The proposed workflow links structured assessment to individualized management and follow-up, but its effectiveness remains untested in this report. Transfer to mood-disorder services is a hypothesis. Such systems require explicit clinical accountability, privacy protection, and human oversight (8).

## Integrating digital therapeutics into person-centered care

**Figure 1** brings these contributions together without implying equal evidence or a validated integrated model. Their shared implication is that intervention selection, engagement, and assessment must be connected within a care pathway. Digital tools should complement psychological and pharmacological treatment where indicated, with shared decisions, named clinical responsibilities, and routes from self-management to professional support. This is consistent with WHO guidance that digital interventions cannot substitute for functioning health systems (9).

**Figure 1:**
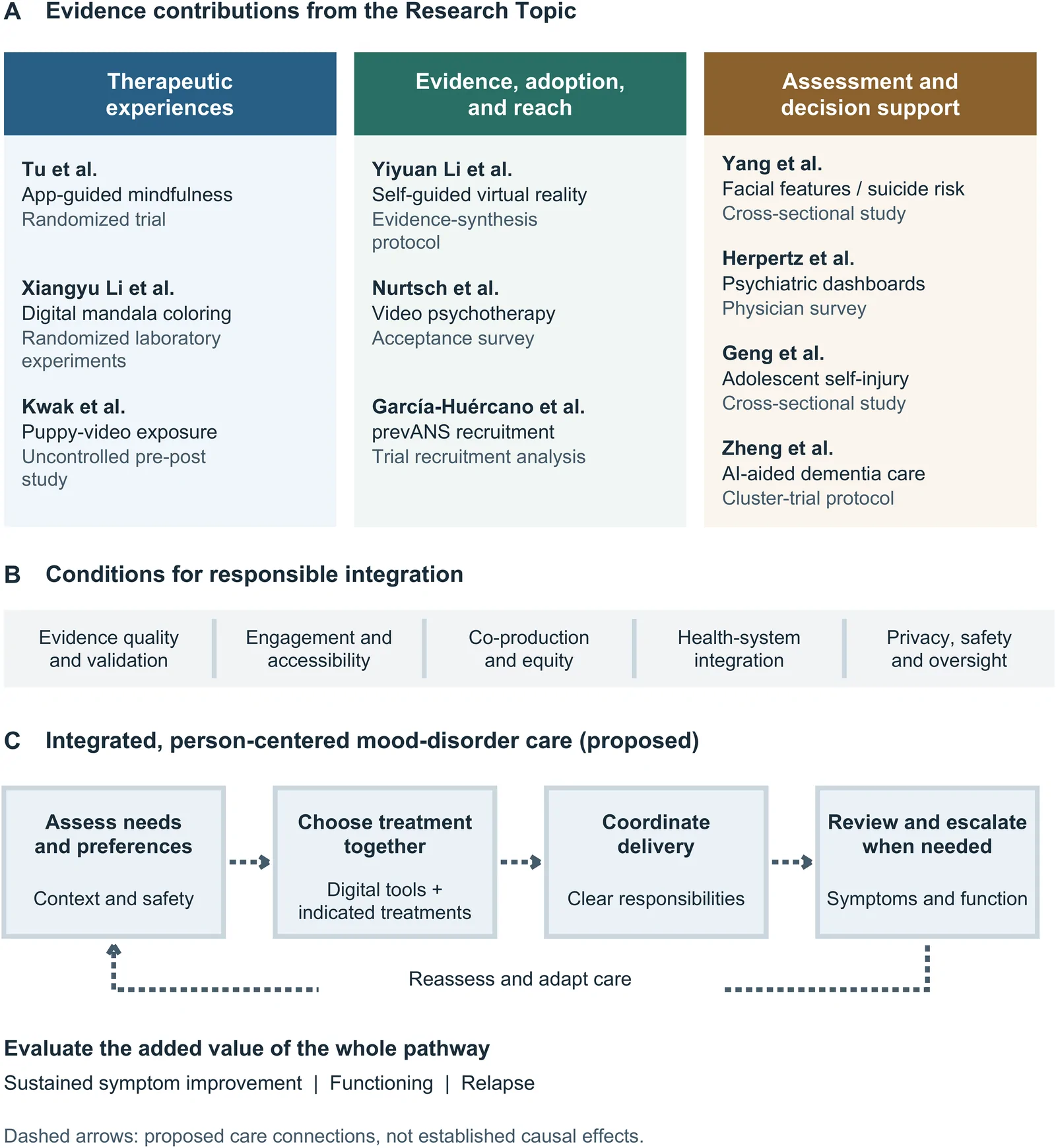
Integrating digital therapeutics into person-centered mood-disorder care. **(A)** The ten Research Topic articles are grouped by contribution, with study designs indicating distinct evidence types. **(B)** Five cross-cutting conditions for responsible integration. **(C)** A proposed pathway links assessment, shared treatment selection, coordinated delivery, and review, with feedback to reassess and adapt care. Digital tools complement other treatments where indicated. Evaluation should test added value for sustained symptom improvement, functioning, and relapse. Dashed arrows denote proposed care connections, not established causal effects. The dementia protocol offers a potentially transferable workflow, not mood-disorder efficacy evidence. This narrative synthesis is not a validated model; the studies do not provide equivalent evidence of clinical benefit. AI, artificial intelligence.

Zhu et al. recently proposed a disease-modification framework for major depressive disorder, including integration of pharmacological approaches with psychotherapy, stress regulation, and digital cognitive training (10). This perspective motivates evaluating digital interventions within longitudinal, multimodal care, rather than as isolated tools. However, neither short-term symptom improvement nor the studies in this collection establish disease modification. Such claims require evidence of altered illness trajectories, alongside functional and mechanistic outcomes.

The next step is to test those connections. Pragmatic comparative trials should evaluate the added value of digital components within coordinated care, prioritizing sustained symptom improvement, functioning, and relapse over longer follow-up. Comparators should distinguish the contributions of therapeutic content, delivery modality, and care coordination. Implementation evaluations should examine differential access and retention, clinician workload, costs, and unintended harms. Co-designed escalation and review procedures should make clear when a digital intervention is insufficient. The collection offers starting points for this agenda; achieving integrated, person-centered care requires evidence that the combined pathway improves outcomes that matter to patients.

## References

[1] Linardon J Torous J Firth J Cuijpers P Messer M Fuller-Tyszkiewicz M . Current evidence on the efficacy of mental health smartphone apps for symptoms of depression and anxiety: a meta-analysis of 176 randomized controlled trials. World Psychiatry. (2024) 23:139–49. doi: 10.1002/wps.21183 38214614 PMC10785982

[2] Amyx M Alebouyeh F Phi NTT Ravaud P Tran VT . Mapping the evidence supporting FDA-authorized digital therapeutics, before and after authorization. NPJ Digit Med. (2026) 9:618. doi: 10.1038/s41746-026-02737-9 42162261 PMC13458057

[3] Borghouts J Eikey E Mark G De Leon C Schueller SM Schneider M . Barriers to and facilitators of user engagement with digital mental health interventions: systematic review. J Med Internet Res. (2021) 23:e24387. doi: 10.2196/24387 33759801 PMC8074985

[4] Brotherdale R Berry K Branitsky A Bucci S . Co-producing digital mental health interventions: a systematic review. Digit Health. (2024) 10:20552076241239172. doi: 10.1177/20552076241239172 38665886 PMC11044797

[5] Singla DR Silver RK Vigod SN Schoueri-Mychasiw N Kim JJ La Porte LM . Task-sharing and telemedicine delivery of psychotherapy to treat perinatal depression: a pragmatic, noninferiority randomized trial. Nat Med. (2025) 31:1214–24. doi: 10.1038/s41591-024-03482-w 40033113 PMC12003186

[6] Spittal MJ Guo XA Kang L Kirtley OJ Clapperton A Hawton K . Machine learning algorithms and their predictive accuracy for suicide and self-harm: systematic review and meta-analysis. PloS Med. (2025) 22:e1004581. doi: 10.1371/journal.pmed.1004581 40934153 PMC12425223

[7] National Institute for Health and Care Excellence . Self-Harm: Assessment, Management and Preventing Recurrence. NICE Guideline NG225 (2022). Available online at: https://www.nice.org.uk/guidance/ng225 (Accessed September 27, 2026). 36595613

[8] World Health Organization . Ethics and Governance of Artificial Intelligence for Health: WHO Guidance. Geneva: World Health Organization (2021). Available online at: https://www.who.int/publications/i/item/9789240029200 (Accessed September 27, 2026).

[9] World Health Organization . WHO Guideline: Recommendations on Digital Interventions for Health System Strengthening. Geneva: World Health Organization (2019). Available online at: https://www.who.int/publications/i/item/9789241550505 (Accessed September 27, 2026). 31162915

[10] Zhu X Lyu N Zhang L Shang L Young AH Wang G . A new therapeutic strategy for major depressive disorder: toward disease modification. Sci Bull. (2026). doi: 10.1016/j.scib.2026.08.029 42660808. [Online ahead of print].

